# Increased Risk of Ischemic Heart Disease in Young Patients with Newly Diagnosed Ankylosing Spondylitis – A Population-Based Longitudinal Follow-Up Study

**DOI:** 10.1371/journal.pone.0064155

**Published:** 2013-05-15

**Authors:** Ya-Ping Huang, Yen-Ho Wang, Shin-Liang Pan

**Affiliations:** 1 Department of Physical Medicine and Rehabilitation, National Taiwan University Hospital Yun-Lin Branch, Yunlin, Taiwan; 2 Department of Physical Medicine and Rehabilitation, National Taiwan University Hospital, Taipei, Taiwan; 3 Department of Physical Medicine and Rehabilitation, National Taiwan University College of Medicine, Taipei, Taiwan; Keio University School of Medicine, Japan

## Abstract

**Background:**

Prospective data is sparse on the association between ischemic heart disease (IHD) and ankylosing spondylitis (AS) in the young. The purpose of this population-based, age- and sex- matched follow-up study was to investigate the risk of IHD in young patients with newly diagnosed AS.

**Methods:**

A total of 4794 persons aged 18 to 45 years with at least two ambulatory visits in 2001 with the principal diagnosis of AS were enrolled in the AS group. The non-AS group consisted of 23970 age- and sex-matched, randomly sampled subjects without AS. The three-year IHD-free survival rate and cumulative incidence of IHD were calculated using the Kaplan-Meier method. The Cox proportional hazards regression model was used to estimate the hazard ratio of IHD after controlling for demographic and cardiovascular co-morbidities.

**Results:**

During follow-up, 70 patients in the AS group and 253 subjects in the non-AS group developed IHD. The cumulative incidence rate of IHD over time was higher in the AS group than the non-AS group. The crude hazard ratio of IHD for the AS group was 1.47 (95% CI, 1.13 to 1.92; p = 0.0043) and the adjusted hazard ratio after controlling for demographic characteristics and comorbid medical disorders was 1.47 (95% CI, 1.13 to 1.92; p = 0.0045).

**Conclusions:**

This study showed an increased risk of developing IHD in young patients with newly diagnosed AS.

## Introduction

Ankylosing spondylitis (AS), characterized by enthesitis of the axial skeleton, is an autoimmune disease with systemic chronic inflammation. [1] AS predominantly affects young subjects, with a peak age of onset between 20- and 30-years-old, and is more prevalent in males. [2]–[4] Cardiovascular manifestations, such as aortic insufficiency, conduction disturbances of the atrioventricular node, and myocardial involvement, are important extra-articular manifestations of this disease. [5], [6] It has been suggested that the cardiovascular manifestations seen in AS patients may result from systemic inflammation and immune-mediated atherogenesis. [7]–[9] However, whether AS patients have an increased risk of developing ischemic heart disease (IHD) is unclear. Several observational studies have shown an increased risk of IHD in AS patients, [10]–[15] but Mathieu et al. [16] and Brophy et al. [17] failed to find a higher rate of myocardial infarction in AS patients. Moreover, most studies evaluating cardiovascular risk were carried out on prevalent AS patients in middle or old age, [11], [12] and little is known about IHD risk in young newly diagnosed AS patients. The aim of this population-based, age- and sex-matched longitudinal follow-up study was therefore to evaluate the risk of developing IHD in young subjects (aged 45 or less) with newly diagnosed AS.

## Materials and Methods

### Data Source

The data used in this study were obtained from the complete National Health Insurance (NHI) claim database in Taiwan for the period 2000 to 2003. The NHI program has been implemented in Taiwan since 1995, and the coverage rate was 96% of the whole population at the end of 2000 and 97% at the end of 2003. It should be noted that the rationale for using the NHI database after 2000 is that, from Jan 1, 2000, according to the rules of the Bureau of NHI, the NHI claim data have been encoded using the standardized International Classification of Disease, 9^th^ Revision, Clinical Modification (ICD-9-CM). To keep individual information confidential in order to satisfy regulations on personal privacy in Taiwan, all personal identification numbers in the data were encrypted by converting them into scrambled numbers before data processing. Because the database used consisted of de-identified secondary data released for research purposes, the study met the requirements of the “Personal Information Protection Act” in Taiwan and was exempt from full review by the National Taiwan University Hospital Research Ethics Committee. The data were analyzed anonymously and the need for informed consent was waived.

### Study Design and Subjects

We used an age- and sex-matched cohort design to study the effect of AS on the risk of developing IHD. The study population included an AS group and a non-AS group, both selected from Taiwanese residents in the complete NHI claim database for 2001, in which more than 21.6 million persons were registered. The Bureau of NHI has formed audit committees to randomly sample the claims data and review charts on a regular basis to verify the diagnostic validity and quality of care.

The AS group consisted of subjects who had received a principal diagnosis of AS (ICD-9-CM code 720 or 720.0) during ambulatory medical care visits between January 1, 2001 and December 31, 2001. The index visit was defined as the first ambulatory visit during which the principal diagnosis of AS was made. To maximize case ascertainment, only patients with at least two ambulatory visits (including the index visit) with a principal diagnosis of AS between January 1, 2001 and December 31, 2001 were considered for inclusion in the AS group (n = 18800). The exclusion criteria for the recruitment of subjects into the AS group were : (1) age less than 18 years (n = 545) or greater than 45 years (n = 6353) to restrict the research sample to the young adult population; (2) a previous diagnosis of AS during year 2000 (n = 6653) to increase the likelihood of identifying only AS cases newly diagnosed in 2001; (3) a diagnosis of any type of IHD (ICD-9-CM codes 410–414) (n = 227) before the index visit; and (4) a diagnosis of diffuse diseases of connective tissue (ICD-9-CM code 710, n = 271) or rheumatoid arthritis (ICD-9-CM code 714, n = 799) before the index visit, resulting in the exclusion of 14006 subjects because of one or more of these criteria. A total of 4794 subjects was therefore included in the final AS group.

The non-AS group was taken from the remaining subjects without a diagnosis of AS in the same 2001 NHI claim database. We assigned the first ambulatory visit during 2001 as the index visit. The exclusion criteria for recruiting subjects into the non-AS group were: (1) a diagnosis of AS before the index visit; (2) a diagnosis of IHD before the index visit; and (3) a diagnosis of diffuse diseases of connective tissue or rheumatoid arthritis before the index visit. We randomly sampled 5 age- and sex-matched persons for each subject in the AS group. A total of 23970 subjects was included in the non-AS group.

### Outcome and Follow-up

All the ambulatory medical care and inpatient records for each subject in the two groups were tracked from their index visit till the end of 2003 and the mortality data for the subjects who died during the follow-up were obtained from the national mortality registry. The date of the first principal diagnosis of IHD (ICD-9-CM codes 410–414) during the follow-up period was defined as the primary endpoint. All subjects were followed from the index visit to the first occurrence of IHD, death, or end of follow-up. We evaluated the effect of AS on IHD-free survival, adjusting for demographic features (age and sex) and the preexisting cardiovascular comorbidities of hypertension (ICD-9-CM code 401–405), diabetes (ICD-9-CM code 250), and hyperlipidemia (ICD-9-CM code 272). Information on comorbid medical disorders was obtained by tracing all the ambulatory medical care and inpatients records in the NHI database in the year before the index visit.

### Statistical Analysis

The Chi-square test and Student’s t test were used to compare differences in demographic characteristics and comorbid medical disorders between the AS and non-AS groups. Incidence rates of IHD were calculated as the number of incident IHD cases divided by IHD-free person-years. The IHD-free survival probabilities for the two groups were estimated using the Kaplan-Meier method. The cumulative incidence was then calculated as one minus the IHD-free survival probability, and differences in cumulative incidence rates between the two groups were tested using the log rank test. Cox proportional hazards regression analysis was used to estimate the effect of AS on occurrence of IHD after adjusting for medical comorbidities (diabetes, hypertension, and hyperlipidemia). Univariate analysis was initially performed for each variable, then the best subset selection method was used to obtain the final multiple regression model. An alpha level of 0.05 was considered statistically significant. The analyses were performed using SAS 9.2 software (SAS Institute, Cary, NC).

## Results

### Descriptive Findings

Table 1 shows the demographic characteristics and medical comorbidities for the AS and non-AS groups. The AS group had a higher prevalence of hyperlipidemia than the non-AS group (p = 0.0214). There was no significant difference between the two groups in the prevalence of diabetes mellitus (p = 0.6639) or hypertension (p = 0.8112).

**Table 1:** Demographic features and comorbid disorders of the AS and non-AS groups.

	Total study population, N = 28764		
Variable	AS group, N = 4794	Non-AS group, N = 23970	p value
Men	3539(73.8)	17695 (73.8)	1.0000
Age, y	31.2±7.6	31.1±7.6	0.2686
Diabetes	75(1.6)	355(1.5)	0.6639
Hypertension	103(2.2)	502(2.1)	0.8112
Hyperlipidemia	125(2.6)	498(2.1)	0.0214

Note: The values are the mean ± standard deviation or the number (%).

### Cumulative Incidence of Ischemic Heart Disease

**Figure 1:**
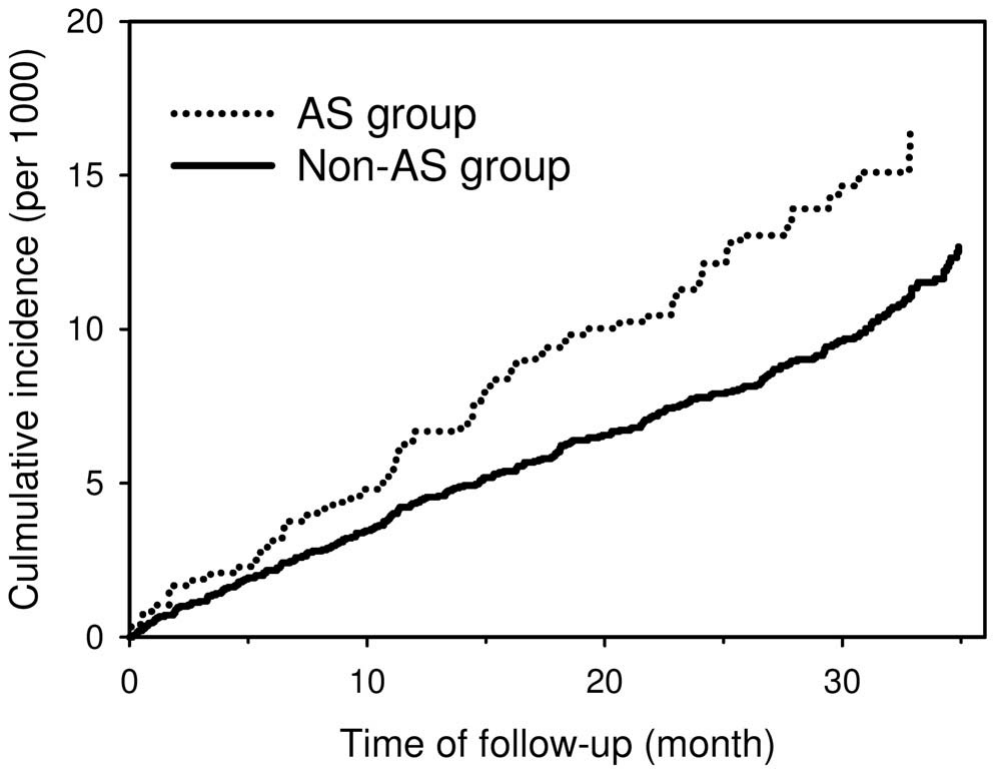
Cumulative incidence of ischemic heart disease (IHD) in the ankylosing spondylitis group (dotted line) and non-AS group (solid line).

The median follow-up time was 31.9 months (inter-quantile range (IQR) = 6.6 months). Of the 4794 patients with AS, 70 developed IHD during 11961.5 person-years of follow-up, giving an incidence rate of 5.8 (95% confidence interval [CI], 4.6 to 7.4) per 1000 person-years. Of the 23970 subjects in the non-AS group, 253 developed IHD during 62337.0 person-years of follow-up, giving an incidence rate of 4.0 (95% CI, 3.6 to 4.6) per 1000 person-years. The cumulative incidence rate of IHD over time was higher in the AS group than the non-AS group (Figure 1, p = 0.0043).

### Cox Regression Analysis

The results of the Cox proportional hazards regression analysis are shown in Table 2. The left panel shows the crude hazard ratio (HR) for each variable based on the univariate analysis. The covariates with a p value less than 0.05 were age, AS, hypertension, diabetes, and hyperlipidemia. Compared to the non-AS group, the crude HR of IHD for the AS group was 1.47 (95% CI, 1.13 to 1.92; p = 0.0043). The middle panel shows the results using the full multivariate model. Age and sex were not included in the multiple regression analysis, since the AS and non-AS groups were matched for these variables. Using the best subset selection method, the final multiple regression model was obtained, as shown in the right panel. The variables included in the final model were AS, hypertension, and hyperlipidemia. The adjusted HR of developing IHD during the 3-year follow-up was 1.47 (95% CI, 1.13 to 1.92; p = 0.0045) for the AS group compared to the non-AS group. Hypertension was associated with a higher risk of IHD (adjusted HR 3.67; 95% CI, 2.39 to 5.64, p<0.0001). The adjusted HR of IHD for hyperlipidemia was 1.53 (95% CI, 0.90 to 2.62, p = 0.1201), which was not significant at an alpha level of 0.05.

**Table 2:** Crude and adjusted hazard ratio (HR) for the occurrence of ischemic heart disease during the three-year follow-up period in the AS and non-AS groups.

	Occurrence of ischemic heart disease					
	Univariate analysis		Full multivariate model		Best subset selected model	
Variable	Crude HR (95% CI)	p value	Adjusted HR (95% CI)	p value	Adjusted HR (95% CI)	p value
Age (year)	1.09 (1.07 to 1.11)	<.0001	NA	NA	NA	NA
Sex (female vs. male)	1.26 (1.00 to 1.60)	0.0531	NA	NA	NA	NA
AS (vs. non-AS)	1.47 (1.13 to 1.92)	0.0043	1.47 (1.13 to 1.92)	0.0045	1.47 (1.13 to 1.92)	0.0045
Hypertension (yes vs. no)	4.08 (2.73 to 6.09)	<.0001	3.56 (2.31 to 5.49)	<.0001	3.67 (2.39 to 5.64)	<.0001
Diabetes mellitus (yes vs. no)	2.30 (1.26 to 4.19)	0.0068	1.40 (0.73 to 2.69)	0.3149	NA	NA
Hyperlipidemia (yes vs. no)	2.35 (1.42 to 3.88)	0.0009	1.42 (0.81 to 2.49)	0.2225	1.53 (0.90 to 2.62)	0.1201

Abbreviations: AS, ankylosing spondylitis; CI, confidence interval; NA, not applicable.

### Sensitivity Analysis

Since ICD code 720 refers to AS and other spondylarthropathies, whereas ICD code 720.0 is specific for AS, we performed sensitivity analysis using a more restrictive case definition to include only subjects with ICD code 720.0 in the AS group. Of the 4794 subjects in the original AS group, the majority (4605, 96%) were diagnosed as ICD code 720.0. The estimated adjusted HR was 1.46 (95% CI, 1.12 to 1.92, p = 0.0058), very close to that obtained in the original analysis (Table 2, adjusted HR 1.47, 95% CI, 1.13 to 1.92, p = 0.0045). In addition, we used another restrictive case definition that included only AS patients who received two principal diagnoses of AS, with at least one being made by a rheumatologist, orthopedist, or physiatrist, and this resulted in an adjusted HR of 1.50 (95% CI, 1.12 to 2.00, p = 0.0058), again very close to the value obtained in the original analysis, suggesting that our findings will hold for different case definitions of AS.

In this study, we used a broad group of ICD-9-CM codes for case definition of hypertension, since physicians may use other ICD-9-CM codes for hypertension patients with a clinical presentation of hypertension-related systemic disease, such as ICD code 401 (essential hypertension), 402 (hypertensive heart disease), 403 (hypertensive renal disease), 404 (hypertensive heart and renal disease), or 405 (secondary hypertension). Since hypertension is a major risk factor of IHD, we investigated the impact of a different definition of hypertension on our findings by restricting the definition to include only ICD code 401 and found that the estimated adjusted hazard ratio of IHD for AS was almost unchanged (adjusted HR 1.47,95% CI, 1.12 to 1.91, p = 0.0047).

## Discussion

The present population-based follow-up study showed that young subjects with newly diagnosed AS were at a higher risk of developing IHD. The three-year cumulative incidence of IHD for the AS group was significantly higher than that for the non-AS group. These findings are consistent with the results from a population-based cohort study using the UK General Practice Research Database that found that men with AS had an increased risk of myocardial infraction compared to men in the general population (adjusted HR 1.44, 95% CI 1.15 to 1.81) [10].

The mechanism responsible for the higher IHD risk in AS patients is unclear, but evidence suggests that it may result from the chronic systemic inflammation seen in AS. [8], [18], [19] Early signs of atherosclerosis, such as increased carotid intra-media thickness, higher carotid pulse pressure, and impaired coronary flow reserve, are more prevalent in AS patients than healthy controls. [20]–[24] Elevated levels of inflammatory biomarkers, such as tumor necrosis factor alpha, C-reactive protein, and interleukin-6, have been found in AS patients. [8], [25], [26] Inflammation is considered to play an important role in endothelial dysfunction and the pathogenesis of arthrosclerosis, [27]–[30], and higher levels of these inflammatory markers have been correlated with an increased risk of atherosclerosis, coronary artery disease, and cardiovascular events. [23], [24], [31]–[33] Thus, AS-related systemic inflammation may be responsible for the higher IHD risk in young subjects with newly diagnosed AS seen in our study.

Previous studies have shown that AS patients have a higher prevalence of hypertension and diabetes mellitus, which may also contribute to the increased cardiovascular risk in AS. [19], [34] However, in our study, there was no significant difference in the prevalence of diabetes or hypertension between the AS and non-AS groups (Table 1). This difference might be explained by the fact that only young subjects with newly diagnosed AS were recruited in our study, whereas most previous studies included all prevalent patients with AS and mainly older patients. Moreover, because the AS and non-AS groups had a similar prevalence of diabetes and hypertension, our findings suggest that AS independently contributes to an increased cardiovascular risk in the young.

In the present population insurance-based study, the estimated prevalence of AS was 0.12% using case definition that requires at least two ambulatory visits with a principal diagnosis of AS in 2001. This prevalence estimate is relatively lower than that obtained from a community-based survey on the prevalence of rheumatic disease in Taiwan [35] which used a 2 stage screening process in 1992. In that study, the estimated prevalence of AS in the adult Taiwanese population ranged from 0.19% to 0.54% [35].

Non-steroidal anti-inflammatory drugs (NSAIDs) are widely used for treating AS, [36], [37] and, since their use has been associated with an increased cardiovascular risk, [38], [39], this raises the possibility that the increased IHD risk in AS patients might result from a secondary effect of NSAID treatment. This was not taken into account in our study, since (i) observational studies on the effects of NSAID use on vascular risks are potentially confounded by indication, as patients with more severe rheumatic diseases are likely to receive higher NSAID doses and to have higher rheumatic disease-related vascular risk and (ii) NSAIDs are widely available as over the counter drugs, so NSAID use is not readily measurable using the insurance database. It is therefore difficult to separate potential adverse effects of NSAIDs from the biological effects of AS itself. Further studies are required to investigate this specific issue.

Since the present study was a large population insurance-based follow-up study and the temporal sequence between AS and IHD was ordered, the observed significant association seems unlikely to be due to selection bias or information bias (e.g. patients with AS would be more likely to be diagnosed as IHD than those without AS). A temporal relationship is essential for establishing a causal connection. Nevertheless, several limitations should be acknowledged. First, the diagnosis of AS, IHD, and medical comorbidities was determined by the ICD codes from the NHI claim database, and information about clinical history, physical examination, and radiographic findings was lacking. This is a major limitation of our study compared to studies using standardized protocols for the diagnosis of AS and there may be concern about the diagnostic accuracy of the database. However, the Bureau of NHI has formed different audit committees that make it a rule to randomly sample the claims data from every hospital and review charts on a regular basis to verify the diagnostic validity and quality of care. Accordingly, the NHI claim database is an established research database and independent studies have demonstrated the validity of the data. [40], [41] Moreover, when restrictive definitions of AS were applied in the sensitivity analyses, the adjusted HR remained almost unchanged, indicating that our findings are likely to hold for various case definitions of AS. Second, although we excluded subjects with a previous diagnosis of AS during year 2000 to increase the likelihood of identifying AS patients newly diagnosed in 2001, it was possible that some prevalent AS cases with more longstanding AS, but coded for the first time in 2001, were included in the AS group. Third, the NHI database lacks some information about lifestyle factors, such as smoking, alcohol consumption, physical inactivity, and obesity, which may affect the interpretation of our results. Finally, since Taiwanese are mainly of Chinese ethnicity, it is uncertain whether our findings can be generalized to other ethnic groups.

### Conclusions

This population-based longitudinal follow-up study shows an increased risk of developing IHD in young patients with newly diagnosed AS and highlights the importance of early risk assessment for IHD in such patients.
